# Evaluation of Unicortical Locking Screw Placement for Torsional Loads in Distal Radius Fractures: A Biomechanical Study in Cadavers

**DOI:** 10.7759/cureus.43522

**Published:** 2023-08-15

**Authors:** Ali T Pehlivan, Bekir E Kilinc, Yunus Oc, Mustafa Vezirhuyuk, Fatih Yamak, Ergun Bozdag

**Affiliations:** 1 Orthopaedics and Traumatology, Denizli Private Health Hospital, Istanbul, TUR; 2 Orthopaedic Surgery and Traumatology, University of Health Sciences, Fatih Sultan Mehmet Training and Research Hospital, Istanbul, TUR; 3 Orthopaedics and Traumatology, Beykent University, Istanbul, TUR; 4 Orthopaedic Surgery, Sanliurfa Training and Research Hospital, Sanliurfa, TUR; 5 Faculty of Mechanical Engineering, Strength of Materials and Biomechanics Laboratory, Istanbul Technical University, Istanbul, TUR

**Keywords:** bi-cortical screw, torsional load, volar plate, uni-cortical screw, distal radius fracture

## Abstract

Background

We aimed to compare bio-mechanical outcomes of short-length 75%-length uni-cortical screw (SL75UCS) and full-length 100%-length screws (FL100S) under axial compression (AXC) and torsional compression (TRC) in cadaveric distal radius volar plate model.

Methodology

A total of 20 wrists from 10 fresh frozen cadavers were included. A 2.5 mm titanium alloy distal radius anatomical plate was placed to the distal radii in full anatomical position, just proximal to the watershed line. Three bi-cortical screws to the shaft of the radius, followed by uni-cortical drilling for distal screwing were placed. Measurement by pulling the drill once it reached the opposite cortex was applied. We selected the screw lengths such that they corresponded to the SL75UCS. In the same configuration for each of the cadavers, we delivered six screws from distal radius holes of the anatomical plate. An oscillating handsaw was used to create an extra-articular distal radius fracture model (AO 23-A3.2). We created a dorsal AP model by performing a 1-cm wedge osteotomy from the dorsal aspect. Complete separation of the volar cortex was achieved. Potting was performed by embedding the shaft of the prepared radius into the polyurethane medium. We placed aluminum apparatus into the distal end to ensure applying of AXC and TRC in bio-mechanistic tests.

Results

No statistically significant difference of stiffness between the SL75UCS and FL100S both under AXC (p=0.88) and TRC (p=0.82). SL75UCS and FL100S groups did not differ in elastic limit under AXC (p=0.71) and TRC (p=0.71). Maximal force on SL75UCS and FL100S groups were also similar under both AXC (p=0.71) and TRC (p=0.50).

Conclusions

Our study findings suggest that drilling the dorsal cortex may not be necessary in the management of distal radius fractures. Instead, utilizing SL75UCS could serve as a viable alternative. This approach offers potential advantages in reducing the risk of extensor tendon complications associated with drilling or screw protrusion. It is a safe method under torsional load to avoid drilling of the dorsal cortex and SL75UCS could be performed in order to prevent from extensor tendon complications secondary to drilling or screw protrusion.

## Introduction

Distal radius fractures are one of the most common types of fractures in orthopedic injuries [1]. While non-surgical approaches are commonly used for treating these fractures in children and adolescents, there is an increasing trend toward surgical intervention in young adults, especially for unstable fractures [2,3]. Internal fixation, which enables early functional rehabilitation, is often the preferred treatment method and can be achieved through various techniques, including fragment-specific fixation, dorsal plating, and volar plating [3-6]. Volar plating, in particular, has become popular due to its ease of application, superior strength, and lower incidence of extensor tendon complications compared to dorsal plating [5].

Injuries to the extensor tendon can potentially occur during the drilling process or postoperatively due to irritation caused by screws protruding into the dorsal surface [7-9]. To address this concern, the use of unicortical delivery of distal screws has been suggested. Previous studies have recommended that the length of screws advanced into the distal part should be 2-4 mm shorter than the calculated length [10]. However, the impact of distal screw length on fixation in volar plate osteosynthesis remains uncertain [8,9]. Previous studies have primarily focused on axial compression (AXC) and bending forces, neglecting the effects of torsional forces (TRC), and have reported similar stabilization rates between short-length (75%-length) unicortical screws (SL75UCS) and full-length (100%-length) unicortical screws (FL100S) [4,11,12].

The hypothesis of our study is that SL75UCS may offer similar stability to FL100S when subjected to both AXC and TRC in the treatment of extra-articular distal radius fractures.

The objective of this study is to compare the biomechanical outcomes of SL75UCS and FL100S in a cadaveric distal radius volar plate model under conditions of AXC and TRC.

This article was previously posted to the medRxiv preprint server on April 27, 2020.

## Materials and methods

The study received approval from the Medical Research Ethics Committee of Acibadem Mehmet Ali Aydinlar University (Approval No: 2019-10/4). A total of 20 wrists from 10 fresh frozen cadavers were obtained from Acibadem University.

**Figure 1:**
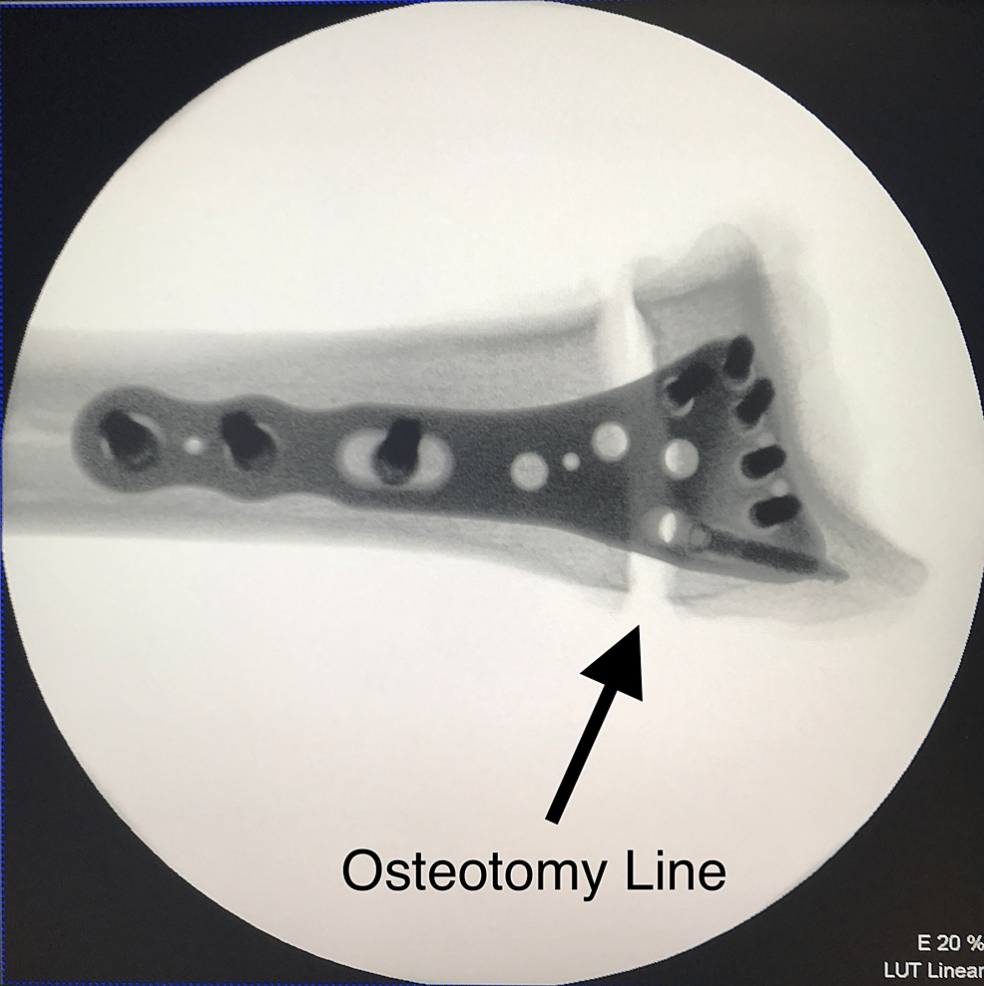
Antero-posterior view of the osteotomized radius with applied plate

**Figure 2:**
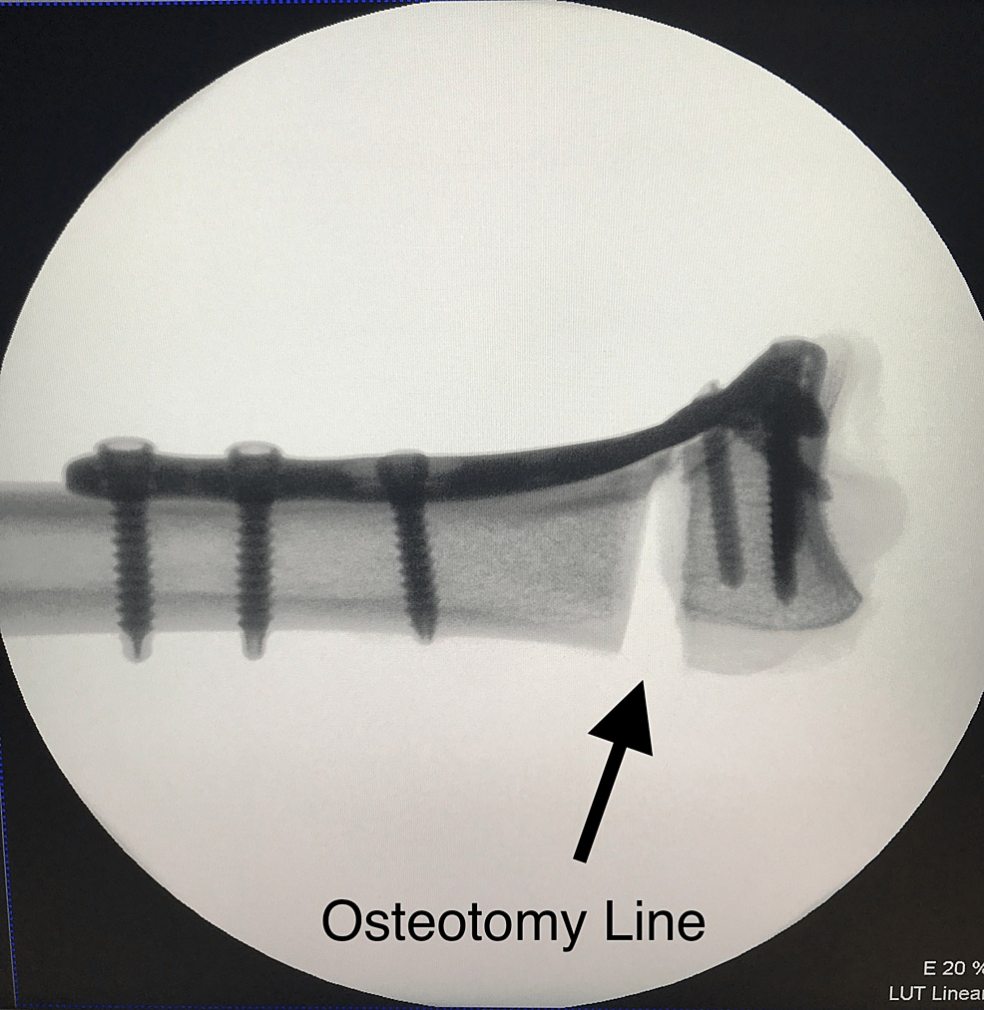
Lateral view of the osteotomized radius with applied plate

Cadavers were included if they did not have any tumors, bone defects or lesions, osteoarthritis, previous fractures, or osteoporosis. We categorized the wrists of the cadavers into homogeneous groups based on dominance (dominant and non-dominant). The radius bones were separated from the ulna and other soft tissues and cut into 14-cm specimens. TST® titanium alloy distal radius anatomical plates were then placed on the volar aspect of the distal radius in their full anatomical positions, just proximal to the watershed line. Following the screw count and configuration suggested by Mehling et al. [13], three bicortical screws were inserted into the radius shaft, followed by unicortical drilling for the distal screws. Measurements were taken by pulling the drill once it reached the opposite cortex. For the distal screws, we selected lengths that corresponded to 75% of the measured length (short-length). Using the same configuration for each cadaver, we inserted six screws from the distal radius holes of the anatomical plate. Subsequently, we created an extra-articular distal radius fracture model (AO 23-A3.2) by performing a 1-cm wedge osteotomy from the dorsal aspect, resulting in a dorsal apex model (Figures 1, 2) [4,14]. Complete separation of the volar cortex was achieved.

**Figure 3:**
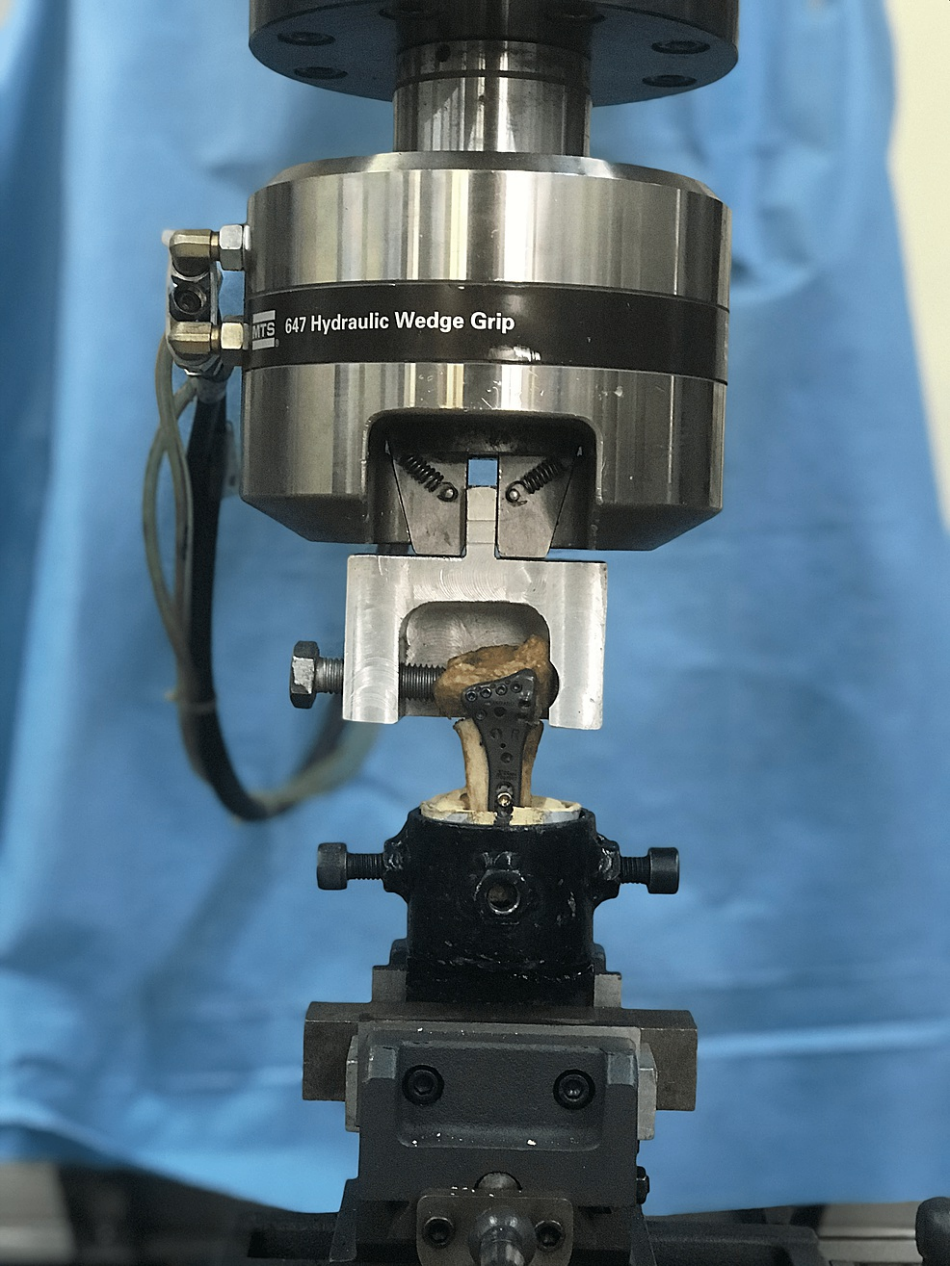
Antero-posterior view of biomechanic test application

**Figure 4:**
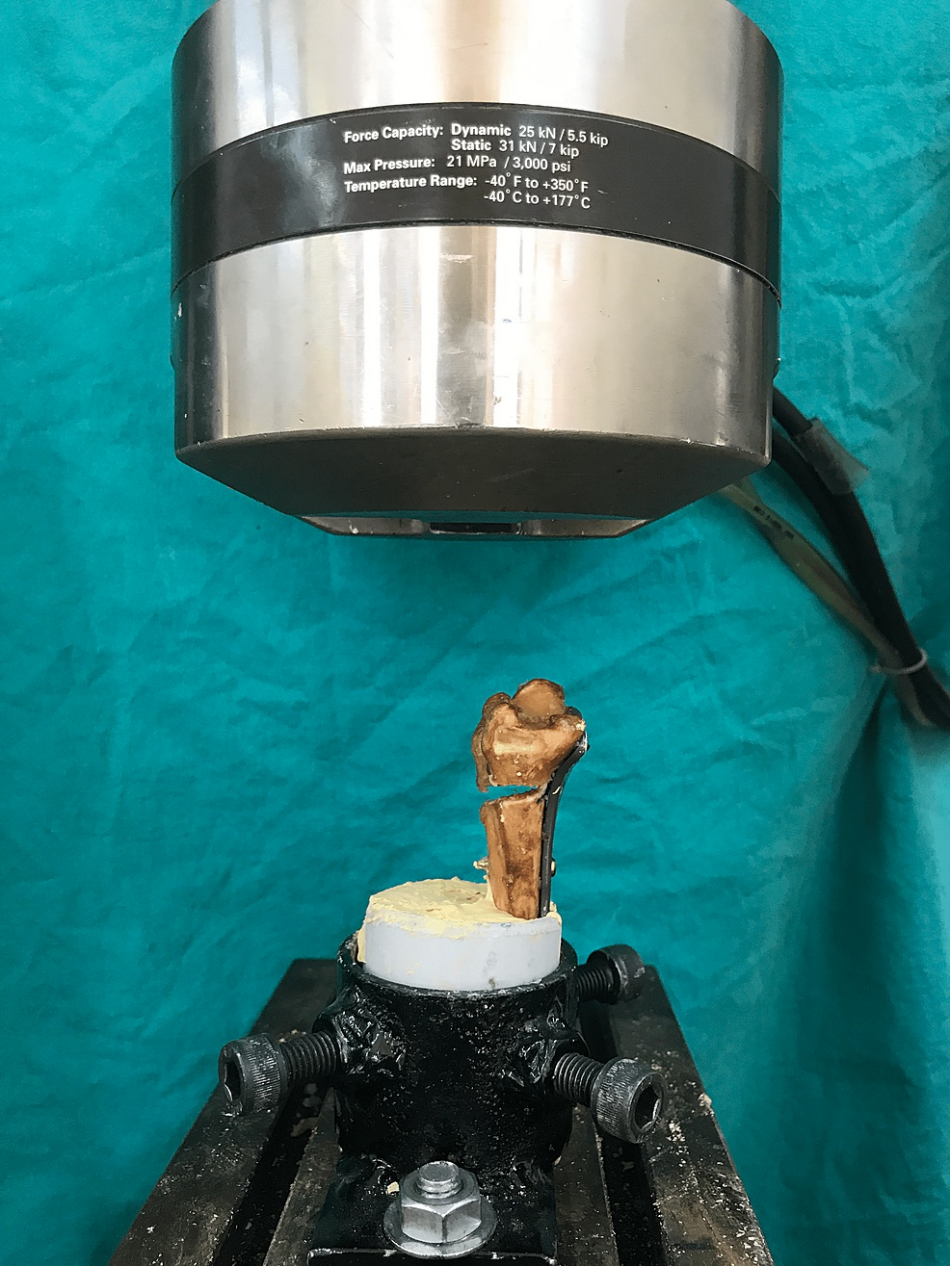
Lateral view of biomechanic test application

The prepared radius shaft was embedded into a polyurethane medium for potting. An aluminum apparatus was placed at the distal end to ensure the application of AXC and TRC during biomechanical tests (Figures 3, 4). All specimens were placed in a testing machine and subjected to AXC and TRC loads. Initially, AXC and TRC were simultaneously applied to each sample, and stiffness and elastic limit measurements were obtained. Subsequently, the maximum force required to cause catastrophic failure (fracture of the bone, screw, or plate) was determined for both the SL75UCS and FL100S groups. The plated cadaveric radius bones were embedded into a polyvinyl chloride tube from one end using polyester resin, while the other end was fixed to the test device via a miniature vise. This vise allowed for both AXC and TRC by clamping the bone perpendicular to the plate plane. A vise was attached to the loading cell (AXIAL-TORSIONAL LOAD TRANSDUCER 25 kN / 25 Nm) of the testing device (MTS 858 Mini Bionix II), and a steel pot was placed in the vise to hold the samples. The prepared samples were placed inside the steel pot using a PVC tube. The upper part of the bone was attached to the test device through the miniature vise. The loads applied to the bone were measured using the transducer (AXIAL-TORSIONAL LOAD TRANSDUCER (2500 N / 25 Nm)), while the displacements and angles were calculated using the displacement transducer (MTS LVDT TRANSDUCER-359/LVDT, Displacement, Serial Number: 10188729) and angle transducer (MTS ADT TRANSDUCER-359/ADT, Torsional Angle, Serial Number: C11382).

After connecting the samples to the device, the test commenced by applying a TRC load ranging from 0.5 Nm to 5 Nm for 10 cycles, while simultaneously subjecting the samples to AXC forces between 5 N and 250 N. The frequency of loading was set at 0.25 Hz to ensure system stability and minimize any gaps. This allowed us to observe the operational range of the system. Subsequently, the loads in the system were reset, and static loading was employed to determine the axial and torsional stiffness of the system, as well as the maximum loads it could withstand. The static tests were conducted with an axial speed of 2 mm/min and a rotational speed of 10°/min. The tests were terminated if there was closure of the osteotomy line or loosening of the screws, as these were considered criteria for damage.

MATLAB 2018 software was utilized to calculate the axial and torsional stiffnesses of the samples under static loadings, their elastic limits, as well as the AXC and TRC moments detected at the moment of fracture.

Statistical analysis

We used Number Cruncher Statistical System 2007 (Kaysville, Utah, USA) software for statistical analysis. Continuous parameters were expressed as mean, standard deviation, median, minimum, and maximum. For the non-normally distributed data, we compared the groups through the Mann-Whitney U test. An overall Type-I error level of five percent was used to infer statistical significance.

## Results

We detected no statistically significant difference of stiffness between the SLS75UCS and FL100S both under AXC (415.8±61.9 N/mm and 410.9±54.2 N/mm, respectively; p=0.88) and TRC (465.0±50.9 N/mm and 456.2±23.8 N/mm, respectively; p=0.82) (Table 1).

**Table 1:** Comparison of the study groups in maximal force under axial and torsional compression

		Total	Short-length unicortical distal screw	Full-length unicortical distal screw	p-value
Axial compression	N/mm, mean ± SD	500.5±37.3	503.0±35.0	497.9±41.2	0.71
	N/mm, median (min-max)	502.5 (424.6-567.8)	511.6 (436.4-556.7)	498.5 (424.6-567.8)	
Torsional compression	N/mm, mean ± SD	5328.2±500.6	5312.2±569.1	5344.1±452.3	0.50
	N/mm, median (min-max)	5213 (4824-6726)	5133 (4824-6726)	5224 (4986-6532)	

## Discussion

Our biomechanical study on a distal radius fracture model established that the utilization of SL75UCS in volar plating offers effective fixation against AXC and TRC forces in the wrist. Through biomechanical measurements, we found that the performance of SL75UCS was comparable to that of FL100S in achieving both AXC and TRC.

Volar plate fixation has gained increasing popularity as a treatment option for distal radius fractures in comparison to dorsal plating. One notable complication associated with the volar plate technique is extensor tendon rupture, which can be mitigated by employing unicortical delivery of distal screws [11]. This method minimizes the risk of extensor tendon injury caused by screws protruding at the dorsal cortex and eliminates direct tendon injury during drilling, as there is no need to penetrate the contralateral cortex. While the advantages of unicortical delivery are well recognized, concerns have been raised regarding its ability to offer sufficient stabilization [6-8]. Prior studies conducted by Wall et al., Liu et al., and Baumbach et al. have reported similar stability between SL75UCS and FL100S in distal radius fracture models, albeit with a focus on axial compression and bending forces rather than torsional forces [4,10,12].

A key objective of volar plate applications is to achieve robust stabilization that facilitates early rehabilitation [10-12]. Throughout the rehabilitation process, the distal radius experiences loads from wrist and finger movements. Although the exact magnitude of these loads in vivo remains uncertain, studies have reported that compression forces induced by wrist movements typically fall below 100 N, with combined compression forces from wrist and finger movements below 250 N [4,10,12]. In our study, the mean maximal force during axial loading was 501 N, while torsional loading reached 5328 N. These findings suggest that the plates possessed a resistance capacity beyond the assumed physiological load and were consistent with relevant studies [4,10]. Several investigations focusing on distal radius fractures have suggested that the assessment of torsional loading serves as a more reliable indicator of stability compared to AXC [6,15-17]. For instance, a study comparing volar plating fixation using locked pegs versus screws alone on sawbones revealed similar outcomes under axial loading but demonstrated superior performance of screws over pegs under TRC [6]. Another biomechanical study on distal radius fractures compared modified double plating with classical double plating and single plating, demonstrating that modified double plating provided improved resistance to torsional loading [15,18]. Notably, our biomechanics study was the first to evaluate the impact of torsional loads on volar plating of distal radius fractures using SL75UCS. Our findings demonstrated that these SL75UCS offered sufficient stabilization against physiological TRC, as well as AXC.

Our study employed fresh frozen cadaver specimens, which differed from the study conducted by Wall et al., who utilized synthetic radius models [4]. To accurately replicate in vivo fractures, we utilized standardized fracture models on cadaveric bones, specifically employing the distal radius fracture model initially described by Baumbach et al. This model has been reported to provide a more realistic representation of fractures compared to previous gold-standard distal radius fracture models [19-21]. Ensuring an appropriate setup for a biomechanical study is crucial to obtain valid outcomes. While studies evaluating shaft fractures of long bones typically allow for sufficient potting area for both proximal and distal segments, our study encountered limitations in fixing fractures that were intraarticular or located close to the joint due to inadequate bone segment availability. However, we designed an aluminum apparatus for the distal bone fragment, enabling the evaluation of rotational loading in conjunction with axial loading.

The number of cadavers used in our study was considered adequate, aligning with the sample sizes reported in the existing literature. Our biomechanical results under AXC and TRC were assessed in relation to established physiological forces exerted on the distal radius [4,10,20,21].

It is crucial to acknowledge the limitations of our study. One of the main limitations is the specific fracture model employed, which, although representing the most commonly encountered distal radius fracture, may have limited applicability to intraarticular fractures, comminuted distal radius fractures, and fractures occurring on the coronal plane. Furthermore, as a biomechanical study, our findings should be further validated through in vivo clinical trials.

Our biomechanical study found that there was no significant difference in the effectiveness of SL75UCS and FL100S when managing extraarticular distal radius fractures with volar locking plate osteosynthesis. These findings indicate that using SL75UCS in volar plating can provide adequate stabilization against physiological AXC and TRC. Importantly, this study is the first cadaveric investigation to demonstrate the satisfactory strength of volar plating in distal radius fractures using SL75UCS against TRC.

## Conclusions

Our study findings suggest that drilling the dorsal cortex may not be necessary in the management of extraarticular distal radius fractures. Instead, utilizing SL75UCS could serve as a viable alternative. This approach offers potential advantages in reducing the risk of extensor tendon complications associated with drilling or screw protrusion. By eliminating the need to penetrate the contralateral cortex and minimizing the potential for screw protrusion, the likelihood of extensor tendon injury can be decreased. However, it is crucial to emphasize that further research and clinical trials are necessary to validate and confirm the benefits of this approach in preventing extensor tendon complications.
